# From emergency myelopoiesis to trained immunity? Reframing post-stroke myeloid reprogramming along the brain-bone marrow axis

**DOI:** 10.3389/fneur.2026.1976078

**Published:** 2026-10-12

**Authors:** TongZhu Wei, YongJiu Wang

**Affiliations:** 1 School of Clinical Medicine, Shandong Second Medical University, Weifang, China; 2 Qingdao Eighth People's Hospital, Shandong Second Medical University, Qingdao, China

**Keywords:** brain-bone marrow axis, emergency myelopoiesis, hematopoietic stem and progenitor cells, ischemic stroke, neuroinflammation, skull bone marrow, trained immunity

## Abstract

Ischemic stroke rapidly mobilizes myeloid cells from anatomically distinct bone-marrow niches, while emerging evidence indicates that severe cerebral injury can also leave a durable imprint on hematopoiesis. These observations have encouraged the use of the term trained immunity in stroke biology, but acute myeloid expansion is not equivalent to innate immune memory. In this Mini review, we distinguish acute myeloid activation, myelopoietic responses consistent with emergency myelopoiesis, and central trained immunity involving hematopoietic stem and progenitor cells (HSPCs). We use an operational four-feature framework—persistence, progenitor involvement, epigenetic or metabolic reprogramming, and functional recall—to grade the strength of current evidence. We consider a sequential transition from emergency myelopoiesis to trained immunity as one testable possibility rather than an established pathway, while allowing for parallel or independent mechanisms. The key unresolved questions are where durable memory is stored, when acute hematopoietic adaptation becomes stabilized, and which outputs are maladaptive versus reparative. Resolving these boundaries is essential before marrow-directed immunomodulation can be translated to patients with ischemic stroke.

## 1 Introduction

Stroke-induced inflammation is now recognized as a systemic neuroimmune response in which the hematopoietic system participates within hours. Adult skull marrow is molecularly distinct from long-bone marrow, cerebrospinal fluid can access calvarial hematopoietic niches, and direct channels provide anatomical routes linking cranial marrow with the meninges and brain surface (1–5). These observations support a specialized brain-skull marrow interface, while anatomical studies show that cranial and vertebral marrow can supply myeloid cells to CNS border tissues (6, 7).

Stroke studies reveal responses across both local and systemic marrow compartments. Ischemia–reperfusion induces lateralized calvarial activation, while single-cell analyses identify distinct myeloid states across brain, skull marrow, femoral marrow, and blood (8, 9). Ischemic stroke also alters the broader marrow microenvironment (10). At later stages, Simats et al. demonstrated months-long myeloid changes, HSPC/chromatin remodeling, and transfer of a pathological phenotype by bone-marrow transplantation (11). These findings indicate that acute marrow activation and durable hematopoietic memory can both occur after cerebral injury, but they do not show that one universally evolves into the other.

Here, acute myeloid activation denotes rapid functional changes in mature myeloid cells without necessarily involving progenitor reprogramming, whereas emergency myelopoiesis refers to demand-adapted myeloid production from HSPCs or progenitors. Trained immunity requires durable altered responsiveness after the initiating stimulus has waned, supported by persistent reprogramming and functional recall; acute expansion or lineage bias alone is insufficient (12–14). This Mini Review therefore asks a focused question: when should post-stroke myeloid reprogramming be interpreted as acute myeloid activation or emergency myelopoiesis, and when is there sufficient evidence for central trained immunity? We explicitly distinguish direct ischemic-stroke evidence from mechanistic analogies in other sterile inflammatory states and treat the proposed continuum as a hypothesis to be tested.

## 2 Acute post-stroke myelopoiesis along the brain-bone marrow axis

### 2.1 Skull bone marrow as a rapid-response neuroimmune niche

The earliest marrow response to ischemic stroke is better viewed as an acute myelopoietic response than as immune memory; demand-driven changes in progenitor output are consistent with emergency myelopoiesis rather than trained immunity per se. The calvaria is a plausible site for this early response because of its distinct molecular profile, resilient hematopoietic capacity, and direct exposure to dural and perivascular signals (1–5).

Stroke-specific studies support this interpretation. Xu et al. (8) identified ipsilateral calvarial proliferation and differentiation, an increased neutrophil-to-monocyte ratio, and osteopontin-dependent meningeal inflammation after ischemia–reperfusion. Yang et al. (9) resolved compartment- and time-dependent myeloid trajectories across calvarial marrow, femoral marrow, blood, and brain. Garcia-Bonilla et al. (15) similarly demonstrated marked divergence of myeloid states between acute and subacute stroke. Together, these studies demonstrate spatially and temporally organized myeloid adaptation but do not generally establish durable progenitor memory or functional recall.

Importantly, the absence of memory evidence does not diminish the extent of acute progenitor remodeling, which can itself accompany emergency myelopoiesis. In non-stroke models, myocardial infarction alters marrow substrate availability, maternal inflammation reorganizes fetal myelopoiesis, early neutrophil-committed progenitors can redirect lineage output, and IL-1α-driven inflammation reshapes emergency hematopoiesis (16–19). Thus, acute progenitor remodeling may be consistent with emergency myelopoiesis but is insufficient to establish central trained immunity without persistence beyond the initiating stimulus and altered responsiveness to a later challenge.

### 2.2 Bidirectional signaling and heterogeneous myeloid outputs

Recent work identifies specific circuits that connect cerebral injury with hematopoietic niches. Kothari et al. (20) described a post-stroke dural-brain pathway in which a mast-cell receptor promotes recruitment of skull-marrow neutrophils and amplifies brain inflammation. In the reverse direction, Menet et al. (21) showed that bone-marrow-derived Dickkopf-1 is progressively induced after ischemic stroke and is sufficient to worsen neurovascular dysfunction and chronic neuroinflammation. Together, these experiments support bidirectional brain-marrow signaling rather than a one-way export of leukocytes.

How brain-derived stress is translated into HSPC decisions remains less certain. A non-stroke neuroendocrine study showed that vasopressin can bias HSC differentiation toward myelopoiesis through a neutrophil-mediated IL-36G-IL-1RL2 loop (22). This is mechanistically informative but should not be presented as an established stroke pathway. The output of marrow-derived cells is also heterogeneous: skull-marrow-derived immune cells can display anti-inflammatory properties after cerebral injury, IL-33/ST2 signaling in monocyte-derived macrophages can preserve early blood–brain barrier integrity, and repair-associated GPNMB/CD63-high macrophages can support angiogenesis and white-matter recovery (23–25).

This heterogeneity prevents a simple equation between myeloid reprogramming and pathology. Stroke can also produce disease-specific myeloid states in distinct CNS compartments, while age changes granulopoiesis and generates atypical neutrophils that worsen microvascular perfusion (26, 27). In parallel, CNS-associated macrophages can restrain leukocyte trafficking in aged animals, and infiltrating monocyte-derived IL-6 can support vascular repair after cerebrovascular injury (28, 29). Thus, the central question is not whether marrow-derived cells are globally harmful or beneficial, but whether a particular post-stroke program becomes durable and functionally recalled.

## 3 From emergency myelopoiesis to trained immunity: where is the evidence?

### 3.1 Operational boundaries and an evidence framework for central trained immunity after stroke

Given this heterogeneity, a more explicit operational framework is needed to distinguish acute responses from durable immune memory. Acute myeloid activation refers here to functional changes in mature myeloid cells, whereas emergency myelopoiesis involves demand-driven remodeling of HSPCs or committed progenitors that alters myeloid cell production (13). Central trained immunity is considered only when altered responsiveness persists beyond the initiating stimulus and is demonstrated by later functional recall or transfer (12, 14). For central/HSPC-mediated trained immunity, we therefore assess four features: (1) post-stimulus persistence; (2) involvement of HSCs/HSPCs or another durable progenitor reservoir; (3) stable epigenetic or metabolic reprogramming; and (4) functional recall after secondary challenge or transfer. Foundational β-glucan, BCG, and Western-diet studies support this framework (30–32).

These features are graded rather than co-equal mandatory criteria. Persistence and functional recall provide the core evidence of immune memory, while HSC/HSPC involvement is required to attribute that memory to the central hematopoietic compartment. Epigenetic or metabolic reprogramming strengthens mechanistic confidence but is treated as missing, not negative, when unmeasured. Studies satisfying all four features therefore provide the strongest support for central trained immunity (12, 30–32). This framework is specific to central training and should not be generalized to all trained-immunity states, because tissue-resident macrophages can acquire local memory without demonstrable HSPC involvement (33, 34).

A further distinction is required between persistent inflammation and genuine trained immunity. Ongoing inflammatory signaling can maintain transcriptional activation without establishing memory. Experimentally, this can be addressed by allowing the primary inflammatory milieu to resolve and then testing whether purified cells or their progeny retain altered responses to a standardized secondary challenge, ideally under stimulus-free ex vivo conditions or after transfer to a naive recipient (11, 31, 32). Persistent epigenetic or metabolic changes strengthen, but do not replace, functional evidence of memory (12, 30–32).

Finally, durable hematopoietic and macrophage programming is biologically heterogeneous. Inflammatory comorbidities and sleep disruption can produce lasting changes in hematopoietic cells, while trained macrophages may occupy distinct metabolic and epigenetic states (35–37). These observations reinforce the need for a context-sensitive standard rather than labeling persistent transcriptional change alone as post-stroke trained immunity.

### 3.2 HSPCs as a memory reservoir: direct stroke evidence and mechanistic analogies

Among ischemic-stroke studies, Simats et al. (11) currently provide the strongest evidence for central trained immunity. Brain ischemia induced monocyte/macrophage states that persisted for months, altered HSPC and monocyte chromatin, and transferred a pathological phenotype through bone-marrow transplantation; IL-1β was a key upstream driver. Together, persistence, progenitor involvement, epigenetic remodeling, and transferability support a hematopoietic memory state rather than sustained activation of mature circulating cells alone. However, generalizability remains uncertain: the study used transient proximal middle cerebral artery occlusion in young mice, with male wild-type cohorts and both sexes in some transgenic experiments, while the small human post-mortem cohort was older, predominantly male, and characterized by severe strokes (11). The human data supported chronic cardiac remodeling but did not directly establish HSPC-mediated memory. Whether comparable memory occurs across other stroke models, severities, age groups, sexes, or comorbidity backgrounds remains unresolved. Aging alters post-stroke granulopoiesis, whereas inflammatory and metabolic comorbidities can reprogram myelopoiesis or HSPCs in non-stroke settings (27, 35, 38). Sex-specific effects on durable post-stroke HSPC memory remain unresolved, despite inclusion of both sexes in some acute/subacute immune-profiling studies (15).

Mechanistic analogies from other inflammatory conditions support the plausibility of HSPC-associated memory but do not substitute for stroke evidence. Heart failure can establish HSC-associated innate immune memory, myocardial infarction can train monocytes through epigenetic regulation of SYK, and pharmacological restraint of HSC activity after myocardial infarction can reduce inflammatory myelopoiesis (39–41). Dietary inflammation can reprogram neutrophil production, while diabetes can transmit persistent epigenetic signals from CD34 + progenitors to inflammatory monocyte progeny (38, 42).

Human studies provide complementary evidence that training-related changes can be encoded in hematopoietic progenitors. BCG vaccination remodels transcription and chromatin accessibility in human HSPCs, and multi-omic profiling identifies epigenetic states associated with later trained responses (43, 44). IL-1β can induce trained features in human hematopoietic progenitors *in vitro* (45). In mice, HSPCs can confer cross-protective memory after transplantation, while CD40-TRAF6 signaling can modify transcriptomic, metabolic, and H3K4me3 features of trained monocytes (46, 47).

Cerebrovascular boundary evidence further illustrates why context matters. High-salt-diet-induced HSPC training impaired reparative macrophage function and worsened recovery after intracerebral hemorrhage (48). Because training preceded hemorrhagic rather than ischemic stroke, this study shows that pre-existing marrow memory can influence cerebrovascular recovery but does not establish that ischemic stroke induces the same program. Likewise, repair-associated macrophages after ischemic stroke demonstrate beneficial myeloid reprogramming, not beneficial trained immunity in the absence of persistence and functional recall (25).

### 3.3 The unresolved relationship: where, when, and with what consequence?

Where? Skull marrow is a plausible site of the earliest lesion-coupled response because of its proximity to the brain, access to cerebrospinal-fluid-derived signals, and regional activation after focal ischemia (1, 2, 8). By contrast, evidence for durable HSPC-associated memory after brain ischemia comes mainly from systemic hematopoietic analyses and bone-marrow transfer, which do not localize the memory reservoir (11). Thus, current evidence does not establish a skull-versus-systemic division of labor. Plausible models include a predominantly acute skull-marrow response with systemic memory, memory in both compartments with different kinetics, or parallel reprogramming of multiple marrow niches.

When? Most direct stroke studies focus on the first 24 h to 7 d (8–10, 20). Simats et al. (11) extend observation to months, but the interval between acute progenitor activation and stable reprogramming remains poorly mapped. Current evidence therefore does not establish emergency myelopoiesis as a necessary precursor of trained immunity; acute myelopoiesis may resolve, or HSPC reprogramming may arise in parallel or partly independently. Non-stroke studies support the plausibility of these alternatives (22, 35, 38). Distinguishing them will require longitudinal HSPC tracking through recovery and subsequent functional recall testing.

With what consequence? Maladaptive versus reparative myeloid outputs appear to vary with upstream signals, host state, local tissue cues, and disease phase. IL-1β-dependent HSPC/monocyte reprogramming and monocyte-derived macrophages have been linked to chronic cardiac inflammation or dysfunction after brain injury and stroke (11, 49). Aging-associated granulopoiesis can generate atypical neutrophils that impair cerebral microvascular perfusion (27). Stroke may also combine systemic innate activation with humoral immune suppression (50). Conversely, IL-33/ST2-dependent macrophage programs, repair-associated macrophages, and monocyte-derived IL-6 can support blood-brain barrier (BBB) integrity, angiogenesis, white-matter recovery, or vascular repair (24, 25, 29). These findings support context-dependent myeloid functions but do not establish a single mechanism governing long-term trained states; reparative outputs should not be considered beneficial trained immunity without persistence and functional recall.

Figure 1 summarizes this temporal–spatial framework and separates maladaptive trained-immunity evidence from reparative macrophage trajectories.

**Figure 1:**
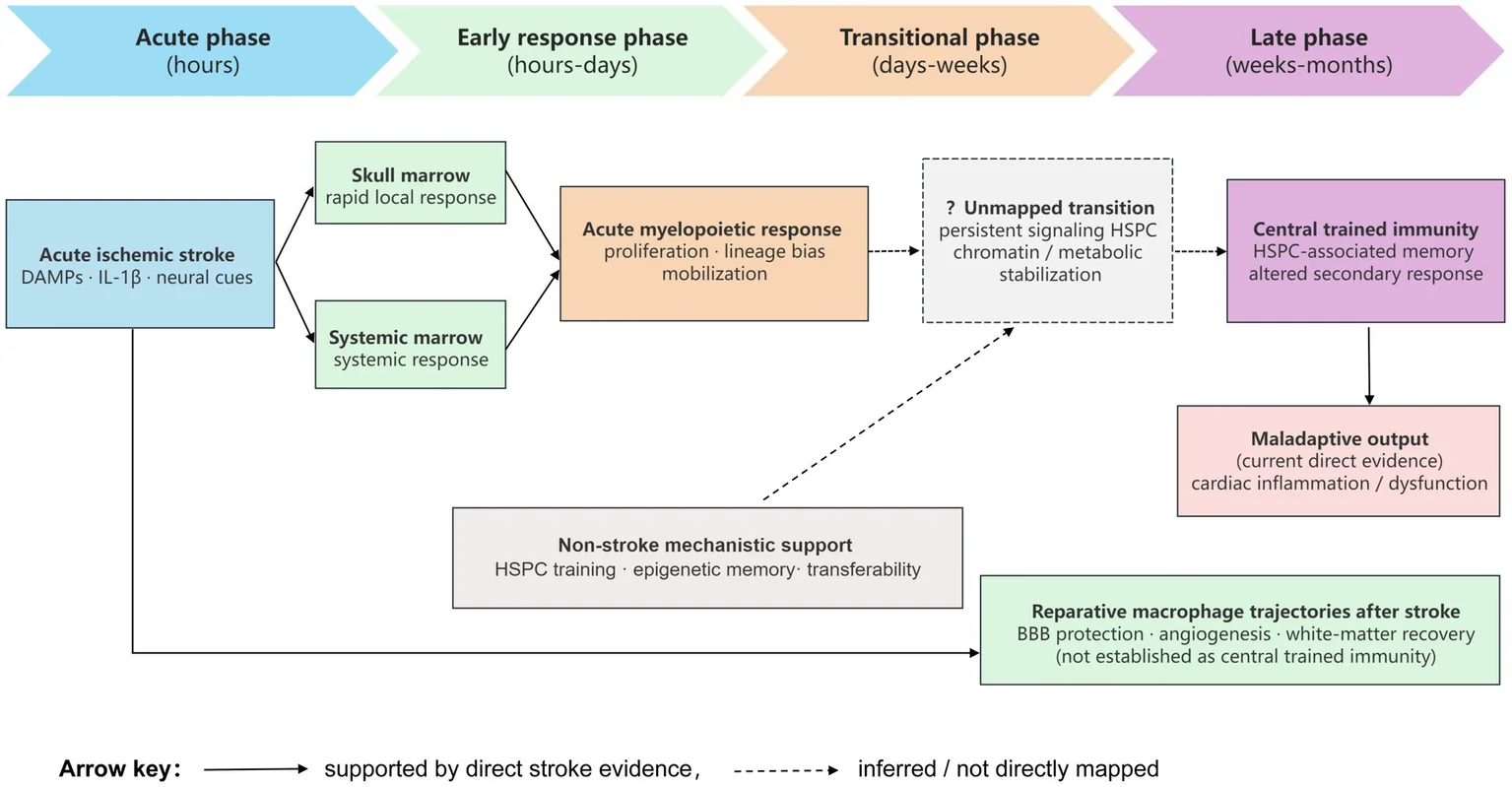
Spatiotemporal framework of post-stroke myeloid responses and potential central trained immunity. Ischemic stroke activates skull and systemic marrow, leading to acute myelopoietic responses. A putative transition to central trained immunity may involve persistent HSPC reprogramming but remains hypothetical. Current direct evidence links HSPC-associated memory with maladaptive cardiac inflammation, whereas reparative macrophage trajectories are shown separately and have not been established as central trained immunity. Non-stroke HSPC studies provide mechanistic support only. Phase labels are schematic. DAMPs, damage-associated molecular patterns; HSPC, hematopoietic stem and progenitor cell.

### 3.4 The human translational gap

The central trained-immunity model remains better established experimentally than in patients with ischemic stroke. Human studies show persistent transcriptional and chromatin-accessibility changes in bone-marrow HSPCs after BCG vaccination, while IL-1β can induce trained phenotypes in human hematopoietic progenitors *in vitro* (43, 45). No longitudinal stroke study has yet linked marrow HSPC reprogramming to later functional recall. Serial blood sampling could nevertheless provide candidate surrogate readouts, including monocyte phenotypes, chromatin accessibility, inflammatory mediators, and standardized ex vivo cytokine responses; longitudinal BCG studies support the feasibility of peripheral epigenetic and functional profiling (44).

A stronger mechanistic design would combine longitudinal blood sampling with bone-marrow analysis in selected cohorts. CD34 + HSPC scRNA-seq/scATAC-seq or targeted chromatin profiling could assess persistent progenitor states, while matched peripheral-cell restimulation could test functional recall; human BCG studies support the feasibility of this approach (43). Because human macrophage memory can be stimulus-specific, both the initial exposure and secondary challenge should be considered when designing recall assays (51). Neither circulating inflammatory markers nor persistent chromatin changes alone should be considered proof of HSPC-associated memory.

Interpretation of these longitudinal readouts is further complicated by baseline factors that can alter myelopoiesis independently of stroke. Aging modifies post-stroke granulopoiesis (27), and diabetes is associated with persistent epigenetic programming in human CD34 + HSPCs (38). Diet-induced obesity can reprogram HSPCs in mice (52), while Western/high-fat dietary states can alter myeloid progenitors and promote atherosclerotic inflammation (32, 42). Infection can also reshape HSC output and innate memory, with effects that vary by pathogen and context (31, 53). Hyperlipidemia may further confound peripheral innate-memory readouts; monocytes from patients with familial hypercholesterolemia have been reported to exhibit a trained phenotype (54). Pre-existing atherosclerotic inflammation should likewise be documented because it may influence baseline innate phenotypes (42, 54). These factors should, where feasible, be incorporated into stratified or adjusted longitudinal analyses rather than attributed to stroke-induced memory.

Table 1 therefore classifies current evidence as direct, supportive, indirect, or insufficient for the specific claim that ischemic stroke induces central/HSPC-mediated trained immunity; these categories reflect evidentiary relevance rather than overall study quality.

**Table 1:** Standardized evidence classification for central/HSPC-mediated trained immunity in studies relevant to stroke.

Study/model	Persistence	HSPC involvement	Epigenetic/metabolic reprogramming	Functional recall/transfer	Evidence class/interpretation
Xu et al. (8) Ischemia–reperfusion; skull marrow	Acute only	Not directly established	Not established as durable memory	No	Insufficient—Acute, spatially organized myelopoiesis; no durable memory or functional recall.
Yang et al. (9) Ischemic stroke; multi-compartment single-cell profiling	Acute/subacute	Inferred progenitor trajectories	Transcriptional states; durable epigenetic memory not shown	No	Insufficient—Acute/subacute myeloid reprogramming; durable persistence and functional recall were not shown.
Kothari et al. (20) Ischemic stroke; dura-skull marrow-brain axis	Acute	No durable HSPC test	No	No	Insufficient—Acute recruitment circuit; no durable HSPC-associated memory assessment.
Menet et al. (21) Ischemic stroke; marrow-derived DKK1	Subacute/chronic signaling	Hematopoiesis affected	No direct durable HSPC epigenetic test	No recall/transfer test	Supportive—Persistent marrow–brain signaling; durable HSPC reprogramming and functional recall were not demonstrated.
Simats et al. (11) Brain ischemia/stroke; long-term follow-up	Yes, months	Yes	Yes; persistent chromatin changes and IL-1β dependence	Yes; bone-marrow transfer	Direct—Persistence, HSPC involvement, chromatin remodeling, and transferability support central trained immunity.
Lin et al. (48) High-salt training before intracerebral hemorrhage	Yes	Yes	Yes; NR4A/OXPHOS-linked training	Yes; marrow transfer	Indirect—Strong HSPC-memory evidence, but training preceded hemorrhagic stroke and was not induced by ischemic stroke.
Zhang et al. (25) Ischemic stroke; repair-associated macrophages	Subacute/recovery phenotype	Not demonstrated as memory reservoir	Metabolic/reparative program	No recall/transfer test	Insufficient—Reparative macrophage program; no durable HSPC-memory or functional recall evidence.

Classes refer to evidence for ischemic-stroke-induced central/HSPC-mediated trained immunity, not study quality. Direct: stroke-induced post-acute persistence, HSPC/progenitor involvement, and functional recall/transfer. Supportive: persistent marrow/progenitor changes with incomplete recall/transfer or mechanistic evidence. Indirect: HSPC-associated memory shown outside ischemic stroke or after pre-stroke training. Insufficient: acute/subacute activation, recruitment, lineage output, or reparative phenotypes without durable memory. Epigenetic/metabolic reprogramming strengthens mechanistic confidence. “No” means not demonstrated, not biologically absent. HSPC, hematopoietic stem and progenitor cell; DKK1, Dickkopf-1; R4A, nuclear receptor subfamily 4 group A; OXPHOS, oxidative phosphorylation.

## 4 Discussion and future directions

Current evidence supports distinguishing acute post-stroke myeloid activation, emergency myelopoiesis, and central trained immunity, but does not establish a fixed sequence among them. Stroke studies demonstrate heterogeneous marrow and myeloid responses (8, 9, 20, 21, 25). Brain ischemia can also produce durable HSPC-associated reprogramming (11). Whether these states occur sequentially or partly independently remains unresolved; Figure 1 therefore presents a testable framework rather than an established pathway. Because direct evidence for durable post-stroke HSPC memory relies largely on a single comprehensive study, its generalizability across stroke models, severities, ages, and sexes remains uncertain (11). Host factors and comorbidities add further uncertainty: aging can alter post-stroke granulopoiesis, while inflammatory or metabolic conditions can reprogram myelopoiesis/HSPCs in other settings (27, 35, 38).

Future studies should compare calvarial and systemic marrow longitudinally and combine lineage tracing with molecular profiling across disease phases. Persistence after resolution of acute inflammation should be coupled with standardized secondary challenge and/or transfer to establish functional recall. Such studies would help distinguish persistent inflammation from durable memory and clarify whether memory is preferentially associated with skull or systemic marrow.

Therapeutically, induction, maintenance, and reversal of maladaptive trained immunity should be distinguished. Induction-directed strategies would target early signals initiating maladaptive HSPC/monocyte reprogramming; IL-1β- and monocyte-trafficking interventions provide proof-of-principle, although the optimal window remains undefined (11). Maintenance-directed strategies would target processes sustaining altered HSPC programs, but stroke-specific mechanisms remain poorly defined. Reversal would require normalization of an established memory state and its recall phenotype, which has not been demonstrated after stroke. Pharmacologic restraint of HSC activity after myocardial infarction illustrates hematopoietic modulation rather than proven reversal of trained immunity (41). Interventions should avoid indiscriminate myeloid suppression and seek to preserve host defense as well as BBB-protective and reparative macrophage programs (24, 25).

In conclusion, whether acute myeloid activation, emergency myelopoiesis, and durable HSPC-associated memory are sequentially linked or partly independent remains unresolved. Defining where memory is stored, how it is maintained, and which outputs are maladaptive versus reparative will help determine whether central trained immunity is generalizable across ischemic-stroke contexts or remains context dependent.
